# Towards ML-Based Diagnostics of Laser–Plasma Interactions

**DOI:** 10.3390/s21216982

**Published:** 2021-10-21

**Authors:** Yury Rodimkov, Shikha Bhadoria, Valentin Volokitin, Evgeny Efimenko, Alexey Polovinkin, Thomas Blackburn, Mattias Marklund, Arkady Gonoskov, Iosif Meyerov

**Affiliations:** 1 Department of Mathematical Software and Supercomputing Technologies, Lobachevsky University, 603950 Nizhni Novgorod, Russia; rodimkov@bk.ru (Y.R.); valyav95@mail.ru (V.V.); 2 Department of Physics, University of Gothenburg, SE-41296 Gothenburg, Sweden; shikha.bhadoria@physics.gu.se (S.B.); tom.blackburn@physics.gu.se (T.B.); mattias.marklund@physics.gu.se (M.M.); 3 Mathematical Center, Lobachevsky University, 603950 Nizhni Novgorod, Russia; 4 Institute of Applied Physics of the Russian Academy of Sciences, 603950 Nizhni Novgorod, Russia; evgeny.efimenko@gmail.com; 5 Adv Learning Systems, TDATA, Intel, Chandler, AZ 85226, USA; alexey.polovinkin@gmail.com

**Keywords:** laser–plasma, machine learning, neural network, dimension reduction, data augmentation

## Abstract

The power of machine learning (ML) in feature identification can be harnessed for determining quantities in experiments that are difficult to measure directly. However, if an ML model is trained on simulated data, rather than experimental results, the differences between the two can pose an obstacle to reliable data extraction. Here we report on the development of ML-based diagnostics for experiments on high-intensity laser–matter interactions. With the intention to accentuate robust, physics-governed features, the presence of which is tolerant to such differences, we test the application of principal component analysis, data augmentation and training with data that has superimposed noise of gradually increasing amplitude. Using synthetic data of simulated experiments, we identify that the approach based on the noise of increasing amplitude yields the most accurate ML models and thus is likely to be useful in similar projects on ML-based diagnostics.

## 1. Introduction

Machine learning (ML) methods open novel ways for solving long-standing problems in many areas of physics, including plasma physics [1,2,3,4], condensed-matter physics [5,6], quantum physics [7,8,9,10], thermodynamics [11], quantum chemistry [12], particle physics [13] and many others [14,15]. One prominent problem for ML methods is to generalize results of numerical simulations, such that they can be used to extract unknown information from experimentally measured data. In contrast to conventional approaches, for which a particular feature in the data is exploited, ML methods are designed to learn indicative features when trained to solve the inverse problem, i.e., determine the input of simulation based on the outcome. One may expect that ML methods are not restricted to learning only the features that can be easily explained and quantified or, at least, this process can be automated to yield higher accuracy and/or broader applicability than that provided by traditional approaches within limited efforts. A particular challenge is how to prevent the ML model from learning and making decisions based on features that are an artifact of synthetic data (results of simulation) only, i.e., on the features that are not present in the experimental data. One general approach is based on adding noise (jitter) to the input data or on varying parameters of extended parameter space [16,17]. The stochastic and/or probabilistic nature of the simulated process can limit the attainable accuracy or make the inverse problem ill-posed, whether globally or locally. In this article, we report on the developments of the ML-based diagnostics for the experiments on high-intensity laser–target interactions, in the area where the difficulty of experimental diagnostics is known to hamper a broad range of theoretically anticipated breakthroughs, ranging from unique XUV sources [18,19] to compact particle accelerators [20,21].

In our study, we employ ML methods for developing diagnostics for experiments on high-intensity laser–matter interactions. In these experiments, a pulse of a few optical cycles in duration is focused to a spot of a few wavelengths in diameter on the surface of a solid target. Here the extraordinarily high intensity of electromagnetic radiation causes ionization of matter and drives relativistic plasma dynamics, causing the transformation of the laser energy into short bursts of XUV radiation or bunches of electrons and/or ions. This conversion happens within just tens of femtoseconds, within a space that is only a few microns in size. Such extremely short spatio-temporal scales, in combination with the complexity and, in some cases, limited reproducibility of experiments, makes it almost impossible to arrange a complete diagnosis of the process. In addition, sufficiently intense foregoing laser radiation (prepulse) can cause the formation and spreading of plasma before the arrival of the main laser pulse (preplasma) in a way that affects the experimental results but is hard to diagnose in-situ. It is also advantageous to use tight focusing that presents particular challenges, in particular, due to the broad spectrum of short laser pulses (see, e.g., [22]). As a result, almost all experiments lack information about what actually happens at the focus and this ultimately hampers the realization of promising applications of laser–plasma interactions.

Although it is possible to train a neural network (NN) to determine interaction parameters from the simulated XUV spectra [2], the sensitivity of the trained NNs to noise [23] or to assumptions inherent in the simulations makes it difficult to extend their use to experimental data. However, the use of simulation results for training is warranted by the much higher cost of gathering experimental data, as well as by the limitations of experimental diagnostics and tunability. There is every reason to expect similar difficulties in other projects on ML-based diagnostics of laser–matter interactions. It has been shown that noise in the supplied dataset can dramatically decrease prediction accuracy for many machine learning algorithms. There are two common approaches to deal with noisy data. In the first approach, algorithms themselves are designed to be robust so they can tolerate a certain amount of noise in the data, without overfitting. Among these are: L1 [24] and L2 [25] regularization; cross-validation [26] or data augmentation [27], to better estimate the generalization error of small noisy datasets; and ensembles of weaker learners, where the overall model tends to be more robust (e.g., random forest [28] and gradient boosted decision trees [29]). Alternatively, noisy data are repaired by replacing the corrupted values with more appropriate ones: the more commonly used techniques are principal component analysis [30], fast Fourier transform and wavelet transform denoising [31], and deep denoising autoencoders [32]).

In this article, we report on the development of ML-based methods for extraction of information that is missing from experimental results, namely, the preplasma scale length and the pulse carrier envelope phase, from the spectrum of XUV radiation generated in the interaction. The spectrum can be both measured in an experiment and obtained via simulation (under certain assumptions). In particular, we focus on making ML models tolerant to the noise in the input data, which improves their generalization capabilities. For this purpose, we use principal component analysis, data augmentation and the addition of noise, with an amplitude that gradually increases over the training process.

The project is arranged as follows. First, we simulate a range of possible experiments and generate synthetic results, each of which is labeled by the values of the parameters that are difficult to measure. Next, we use this dataset to train neural networks to solve the following regression problem: reconstruct the unknown parameters from the simulated measurements. Next we apply principal component analysis to see whether the use of a truncated vector of leading components can increase the accuracy and/or reduce computational demands. We also test the use of data augmentation, based on superimposing random noise. After that, we consider the problem of making the ML model tolerant to the noise in real experimental data. For this purpose, we try increasing the amplitude of noise over the training process.

We note that there is a conventional approach of spectral interferometry that can be used to retrieve information about relative intervals between few XUV peaks generated in the interaction process [33]. Our intention is to identify whether the ML-methods can be used to extend the capabilities of these diagnostics.

## 2. Problem Statement

To set the context, we begin with a brief overview of the physical process to be diagnosed using ML, as well as of the methods used for its description and simulation. The process in question is the interaction of an intense laser pulse with dense plasma that emerged from the ionization of a target. For the conditions of interest, the temporal evolution of this system can be described by Maxwell’s equations for the electromagnetic field and by the relativistic equation of motion for plasma particles.

In our work, the numerical simulation of the laser–plasma interaction is based on the Particle-in-Cell (PIC) method. Here, we briefly describe the method; a detailed description is given in [34]. The PIC method is used to model interaction of an electromagnetic field with plasma using kinetic description. This method operates on two distinct sets of data that correspond to the electromagnetic field and plasma particles. Values of electric and magnetic fields are defined on a spatial grid. Plasma is represented as an ensemble of particles, each with a charge, mass, position and momentum. Each particle of the ensemble represents a cloud of real particles. The distribution of real particles in each cloud is given by a form factor function. A notable feature of the method is that particles do not interact with each other directly; instead the form factor is used to compute a cumulative current (at grid nodes) that changes the electromagnetic field acting on the particles. The conventional computational loop of the PIC method consists of four stages. Field values are updated by solving Maxwell’s equations

(1) $$\frac{\partial E}{\partial t}=c\nabla \times B-4\pi J$$

(2) $$\frac{\partial B}{\partial t}=-c\nabla \times E$$

where E and B are the electric and magnetic fields, respectively, J is the current density produced by particles in motion, *c* is the speed of light. These equations can be solved using FDTD [35] or FFT-based [34] techniques. For each particle, the Lorenz force is computed using interpolated values of the electromagnetic field and the particle momentum and position are updated. The grid values of the current created by the motion of particles are computed and added to Maxwell’s equations forming a self-consistent system of equations. The equations of motion are solved together with the relativistic form of Newton’s second law. The system of equations can be written as

(3) $$\frac{dr}{dt}=v$$

(4) $$\frac{dp}{dt}=q\left(E+\frac{1}{c}v\times B\right)$$

(5) $$p=\frac{mv}{\sqrt{1-\frac{v^{2}}{c^{2}}}}=\gamma mv$$

where r, v, p are the position, velocity and momentum, m, q are charge and rest mass, γ is the Lorenz factor, respectively.

One interesting goal in laser–plasma interaction physics is to achieve a specific interaction regime [36], which was theoretically revealed to yield unprecedentedly strong bursts of XUV radiation for fundamental and applied science as well as for non-destructive compact diagnostics in industry [19,37,38,39,40,41]. Although the needed regime is well studied with simulations and theoretical insights [42], achieving it in an experiment requires matching of difficult-to-measure/control interaction parameters, such as the pre-plasma scale length and the shape of the laser pulse at the focus. The routinely measurable spectra of outgoing radiation are likely to contain the information needed for educated adjustment of the experiment, but the complexity of the process makes it difficult to develop theory-guided strategies for this. As of now, the state-of-the-art developments concern the use of spectral interferometry for coincidence (verification) assessment under certain restricted conditions [33], whereas the lack of more generic diagnostics methodology is recognized as an essential stumbling block for further developments in this area.

As is the case in many other research areas, ML-based methods have recently been recognized as a way to resolve long-standing problems in laser–plasma physics [2,3,4]. ML methods are known to provide a generic way to solve inverse problems, which in our case means learning the interaction parameters from the spectrum, whereas training can be arranged based on the results of simulations or analytical models. This, however, poses several questions: Whether the indicative features are indeed present in the spectra? How accurate can the solution be? How do we make ML methods learn features caused by physics rather than by numerical aspects and not lose reading capabilities when applied to real experimental data? For particular parameters, it was shown that the NN can learn features in analytical spectra and then identify interaction parameters of simulated experiments [2]. In the present study, we try to determine the limits of this methodology, understand the limiting factors and identify how they can be alleviated.

## 3. Data Acquisition

To acquire data for training, we numerically simulate the process of a laser irradiating plasma target using PIC simulations with the ELMIS code [43]. A series of simulations has been performed where a p-polarised laser pulse with wavelength $\lambda_{0}\;=$ 765 nm and peak intensity of $I_{0}\;=$ $8.9\times 10^{19}$ W/cm^2^ (equivalent to normalized amplitude $a_{0}=eE_{0}/m_{e}\omega_{0}c\approx 6.1659$) irradiates a $Si^{4+}$ plasma at an angle of incidence θ. Here $E_{0}$ is the peak electric field strength of the laser pulse, $e,\;\omega_{0}$, $m_{e}$ and *c* are the electron charge, the laser frequency, the electron mass and the velocity of light in vacuum. The laser is incident on the plasma at an angle θ, and this oblique problem is reduced to one dimension by the Bourdier technique [44], where a frame boosted by csinθ along plasma surface is chosen. In this reference frame, the incident laser pulse is described by the following profile

(6) $$\begin{array}{c} E_{y}=E_{0}^{\prime }\Theta \left(\psi \right)\Theta \left(4\pi -\psi \right)\sin\left(\psi /4\right)\times \;\;\;\;\;\;\;\;\;\;\;\;\;\;\;\\ \;\;\;\;\;\;\;\;\;\;\;\;\;\;\;\;[0.5\cos\left(\psi /4\right)\sin\left(\psi \right)+\sin\left(\psi /4\right)\cos\left(\psi +\phi_{\mathrm{CEP}}\right)] \end{array}$$

and $\psi =2\pi \left(x-ct\right)/\lambda^{\prime }$, where *x* and *t* are the coordinate (normal direction towards the target) and time, $E_{0}^{\prime }=E_{0}\cos\theta$ is the wave amplitude in the moving frame, Θ is the Heaviside step function, $\phi_{\mathrm{CEP}}$ is the carrier envelope phase of the laser pulse, $\lambda^{\prime }=\lambda_{0}/\cos\theta$ is the wavelength of the laser in the moving frame.

The initial temperature of the plasma is 1 keV and the ions in the plasma are mobile, with mass ratio $m_{i}/m_{e}=28\times 1836,$ ($m_{i}$ being the mass of the ion). The plasma slab (with a step function profile) has a width of 2 μm and a peak density of 400 $n_{c}$, where $n_{c}$ is the classical critical density for the laser to reflect off the plasma ($n_{c}=m_{e}\omega_{0}^{2}/4\pi e^{2}$). This is preceded by an exponentially decaying preplasma with a scale length $L_{p}$. The length of the simulation box is $L_{x}=16\lambda^{\prime }$ with spatial resolution $\Delta_{x}$ between 0.24 nm for $0^{\circ }$ and 0.47 nm for $75^{\circ }$, small enough even at larger angles to avoid numerical instabilities. The number of particles per cell are 85, and there are 256 steps per plasma period.

The simulations span the following parameter space: the angle of incidence,

(7) $$\theta \in [0^{\circ },75^{\circ }],$$

with a step size of 2.5°, the carrier envelope phase

(8) $$\phi_{\mathrm{CEP}}\in \left[0^{\circ },180^{\circ }\right]$$

with a step size of $6^{\circ }$ and the pre-plasma scale length is varied from

(9) $$L_{p}\in \left[0.08,0.73\right]\mu m,$$

with a step size of 0.05 μm. This amounts to a total of 14,415 simulations.

**Figure 1:**
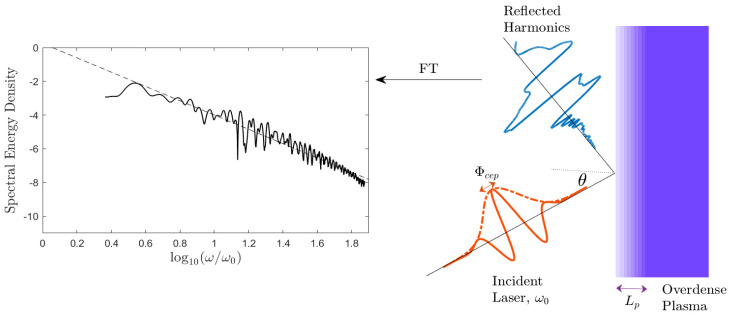
A schematic diagram showing the simulated scenario. A laser pulse angularly incident on a solid plasma target with a pre-plasma length. The reflected spectrum contains harmonics of the laser’s initial frequency, and the intensity of these harmonics decays with increasing harmonic order.

After the laser is reflected off the dense impenetrable plasma, the spatially varying electric signal is computed by the PIC code, which contains the information about one laser–plasma interaction scenario. This is Fourier-transformed to yield the spectral (i.e., frequency-resolved) energy density of the radiation. Thus, for each *i*’th numerical simulation with laser–plasma configuration given by a vector of parameters $y_{i}=\left(\theta^{i},\Phi_{CEP}^{i},L_{p}^{i}\right)$, there is a corresponding *i*’th spectrum that is referred to as *i*-th object or *i*-th sample in the following text. The setup is summarized in Figure 1, where an example of spectrum is shown.

## 4. Experimental Results

### 4.1. Metrics

To assess the quality of ML models in our study, we compute the Mean Absolute Percentage Error (MAPE) and the coefficient of determination ($R^{2}$):

$$\begin{array}{c} MAPE=\frac{100}{n}\sum_{i=1}^{n}\frac{|\hat{y_{i}}-y_{i}|}{\max(\hat{y})}, \end{array}$$

$$\begin{array}{c} R^{2}=1-\sum_{i=1}^{n}\frac{{\left(\hat{y_{i}}-y_{i}\right)}^{2}}{{\left(y_{i}-\bar{y}\right)}^{2}}, \end{array}$$

where $\hat{y_{i}}$ and $y_{i}$ are the true and the predicted value of the *i*-th object, respectively, and $\bar{y}$ is the value of the predicted parameter averaged over the training set.

In all the experiments described below, we employed a 85/15 train/test split. The accuracy was averaged over several runs with random distribution of samples between training and test sets. Namely, we trained the NN 3 times to find the optimal number of principal components, and 10 times in other experiments. The training process was rather stable: the accuracy of the model from each individual run differed from the average accuracy for no more than 10%. For labels, the transformation has linear scaling, whereas for the features (spectral energy density values *y*), we first compute the logarithm, determine the range of values for each frequency and linearly scale it to [−1;1]. The metrics were calculated after performing the inverse transformation, that is, in the original scales.

### 4.2. Methods

The experimental part of the paper is as follows. In the beginning, we train different fully connected NN-s without any data preprocessing (except for transforming the spectral energy density values to the range [−1;1]) and choose the best architecture in terms of proposed metrics as a baseline model (we vary the number of layers and the number of neurons in each layer). In order to improve the accuracy of this baseline model, we try different approaches. First, we apply the principal component analysis (PCA) to reduce the number of input features, assuming that this procedure may facilitate the extraction of useful information from the input spectra, simplify the training process and finally lead to more accurate predictions. Second, we employ a widely used data augmentation technique [45,46] to increase the size of the training set. In our case, the data augmentation is limited only to adding Gaussian noise to spectral data, because other possible methods (shift, extension, dilation) are irrelevant for the data in question. The noise is added only to the training samples, while the inference is performed on the clean data.

Another reason for adding the noise is to develop the tolerance of the NN to the non-systematic, stochastic variation of harmonic intensities in the high ends of the spectra obtained from the numerical simulations. Our ultimate goal is to apply a similar approach to extract information from real experimental data. We expect that the data obtained from future experiments will differ from the simulated spectra and our ML model must be tolerant to this difference. As a first step on this way, we assume that the experimental data can be much noisier, so we try to find methods to train the NN to extract useful information from such data by adding Gaussian noise to the clean data. In contrast to the augmentation procedure described above there are two main differences: first, the amplitude of noise is not uniform but is determined based on the data set. Second, the noise is added to both the training and the test sample. We use a similar procedure as before: we train the baseline fully-connected NN and then try to improve its quality. First, we try to decrease the model input using PCA and, finally, we train the model on the initial data gradually increasing the noise.

In all the experiments we use Keras and Tensorflow to train fully connected NN-s, and Scikit-learn to perform PCA. All the values of the parameters needed to reproduce the experimental results are presented below in the following subsections. All the scripts and datasets are publicly available [47].

### 4.3. Experiments with Data of Numerical Simulation

#### 4.3.1. Baseline Model

A fully connected network was chosen as the basic option for reconstructing the parameters based on a given spectrum. We tried models with a different number of layers and neurons and chose the model with 7 hidden layers and the following number of neurons: 1024, 512, 256, 256, 128, 32, 16, correspondingly. We used the ReLU activation function and the Adam optimizer with the learning rate equal to $5\times 10^{-4}$. We trained the model for 400 epochs with a batch size of 128. After 300 epochs the learning rate was multiplied by 0.99 every epoch to fine-tune the parameters of the NN when approaching the local minimum, as well as to prevent leaving the neighborhood of the local minimum.

**Figure 2:**
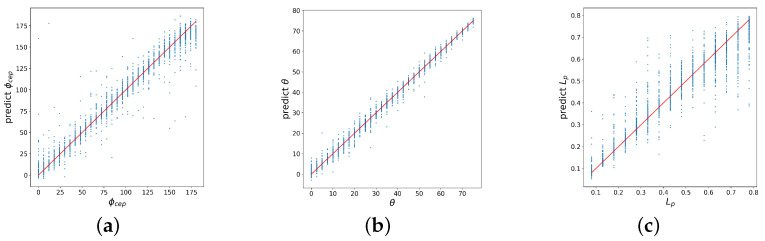
Correlation of the exact and predicted values of laser–plasma interaction parameters, reconstructed by a baseline fully connected NN trained on a clean data: (**a**) carrier envelope phase, (**b**) incidence angle, (**c**) pre-plasma length.

The computed averaged accuracy is shown in Table 1. It can be seen that in general the incidence angle θ is reconstructed with an accuracy better than 98%, while the other two parameters are reconstructed with less accuracy. We observe (see Figure 2) that the carrier envelope phase $\Phi_{CEP}$ values are more scattered than θ values, there are also several points with large errors in magnitude. A small bias from the true values can be observed at the boundaries of the considered range of values. The largest error is observed for the pre-plasma length $L_{p}$. It can be seen from Figure 2 that for large values of $L_{p}$, a bias appears in the predictions relative to the correct answer, and the variance of the prediction increases substantially. This can be explained in the following way. The $L_{p}$ parameter means the characteristic length of the exponentially decaying pre-plasma. At large values of $L_{p}$ the plasma density varies with distance slowly and thus the specific value of $L_{p}$ may become hardly distinguishable. In contrast, at small values of pre-plasma length, behavior changes more evidently with its change, so it is much easier for the model to differentiate between different values of $L_{p}$.

The achieved level of accuracy is considered useful for the intended experimental needs and looks reasonable in terms of the observable difference between the spectra obtained at the neighboring locations of the parameter space. The main obstacle for a better differentiation of the cases seems to be the stochastic nature of the simulated process, which results in non-systematic variation of spectral curves especially in the high-frequency range. Our intention for the studies presented below is to identify whether the obtained results can be significantly improved by generic ML methods so that the most useful approaches can be incorporated into our further studies and developments.

#### 4.3.2. PCA Preprocessing

**Figure 3:**
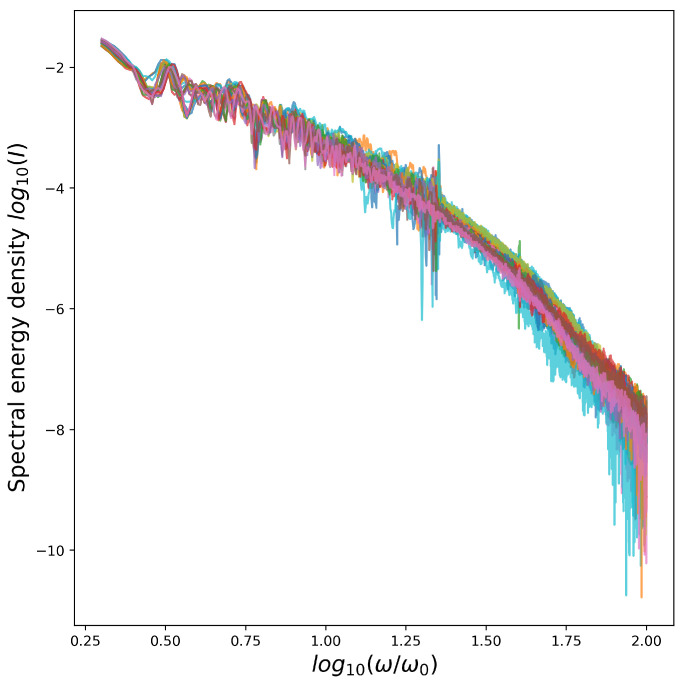
Neighboring spectra for spectrum with the parameters $\Phi_{CEP}=102$, θ=0.25, $L_{p}=0.33$. *I* is the spectral energy density given in a.u.

In the problem under consideration, the energy spectra used as the input contain rapid non-systematic variations at the high-frequency part, see Figure 3, i.e., the values may undergo rapid changes even with a small change in frequency and/or for a small change of input parameters. This is a natural consequence of stochastic factors that are adherent to both simulations and real experiments. One way to restrict the ML model to learn only the most robust features is to use PCA. We can see it as a way to reduce the size of the input vector (keeping the same the size of the training set) or, alternatively, as a way to remove the part that has the least systematic behavior. In both senses, this is what can be useful in our case.

**Figure 4:**
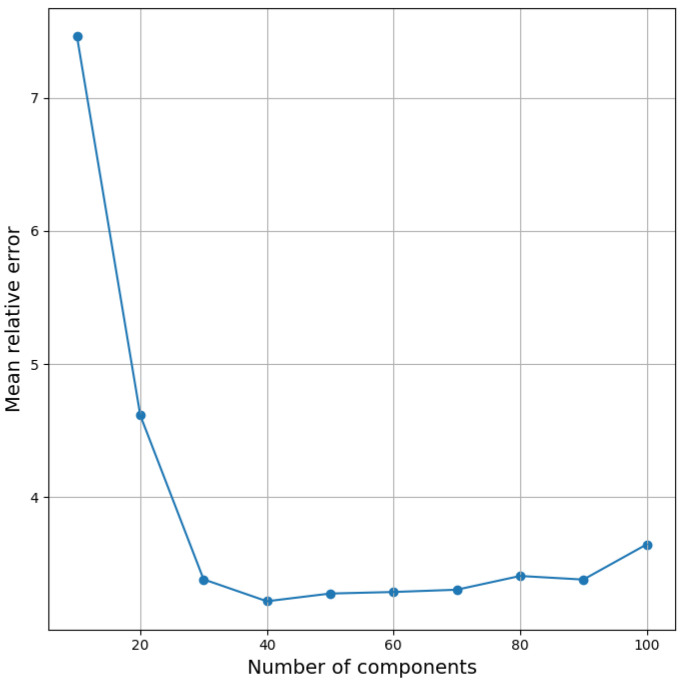
Dependence of the mean relative error averaged over three runs on the number of principal components.

In order to deal with this problem, we preprocessed computed spectra using PCA to reduce their size to a reasonable number of components containing the major part of information. Then we used resulting features as an input of a fully connected NN. First, we determined the optimal number of principal components for the fixed neural net architecture by training NN with a different input size. As a starting point, we took the baseline network with all hyperparameters the same, except a number of hidden neurons in different layers. Since the size of the input data is much smaller after using PCA than the original dimension of the data, we adjusted the first two layers of the network. The resulting NN contained seven hidden layers of 512, 256, 256, 256, 128, 32, 16 neurons, respectively. Before using PCA, each feature was standardized. After using PCA, new features were normalized in the range [−1;1]. To assess the quality of a model trained on a fixed number of components, training was run three times with different split into training and test samples. Based on these experiments, the mean value of the relative error was calculated for each model. Figure 4 shows the results of the selection of the number of principal components. We observe that the choice of 40 principal components is a reasonable compromise between reducing the number of components and maintaining the accuracy of the model for a fixed architecture of the NN. The accuracy of reconstructing the three parameters of the model after averaging over 10 runs is shown in Table 1.

General heuristics exist for determining the number of principal components [48,49,50], which we can compare with the results of exhaustive searches on a given problem. This allows us to identify which heuristics are most reliable and, therefore, which we shall give preference to. Success is not, of course, guaranteed, but it can reduce computational costs. We considered the Kaiser, broken stick, and condition number criteria. The Kaiser criterion shows that it is necessary to take 88 principal components. At the same time, it is known that this criterion is often overestimated. The broken stick criterion indicates 10 principal components, and it is also known that this criterion often underestimates the number of principal components. In addition, the third criterion based on eigenvalues indicates two principal components. This effect arises due to the fact that in this problem the first two principal components retain a large proportion of the data variance. Hence, it follows that for further purposes, one can use the first two heuristics for an initial estimate of the number of principal components or estimate on the basis of the average value of these two criteria in problems with a similar formulation.

As a result, the accuracy of the used ML model is improved, as shown in Table 1. At the same time, problems similar to those observed for the model trained on full spectra remained. The carrier envelope phase $\Phi_{CEP}$ and pre-plasma length $L_{p}$ still have a determination coefficient substantially less than one, which indicates the presence of problems with reconstruction. Thus, the use of the principal component method allows us to reduce the number of features and the size of the network, increase the model accuracy but does not completely remove the previously described problems.

#### 4.3.3. Data Augmentation

Data augmentation is one of the typical options for improving the accuracy of machine learning models by increasing the size of training dataset. Many of the widely used techniques, such as rotations, flips, distortions, used, for example, in computer vision, are not applicable here, since they are clearly irrelevant to the data in question. At the same time there are no probabilistic choices involved in the simulations, i.e., for a fixed set of parameters, spectra look the same. However, the probabilistic generation of initial electrons’ velocities in PIC simulations and, more importantly, the stochastic nature of the process itself causes non-systematic microscopic changes that result in rapid variation of spectral intensity in the high-frequency range. We can mimic this by adding random noise to computed spectra, thus producing more training examples with correct underlying physics. By keeping the level of this noise small, we can try to ensure that such approach will not prevent the neural net from learning the systematic dependency of spectra on the parameters.

In this case, the data augmentation was performed in the following manner. Gaussian noise with zero mean was superimposed on all elements of the training sample spectrum. The standard deviation for each frequency was defined as two percent of the absolute value in the spectrum at that frequency. This procedure was repeated five times for each spectrum to create copies with additional noise. Thus, the volume of training data has increased five-fold. Based on our experiments, a higher number of copies with noise or a higher/lower percentage of noise does not improve the result. Test samples were not augmented and inference on test data was performed on clean data. The network architecture and optimizer parameters were the same as for the baseline network described before, except the number of epochs, which was equal to 200, and the learning rate, which was multiplied every epoch after 150 by 0.95. The averaged result over 10 runs with a random split for the training and test set are shown in Table 1.

As a result of data augmentation, the accuracy of parameter reconstruction increases, which is the expected result, because the training set becomes significantly larger. At the same time, the accuracy improvement is not substantial and the same problems with pre-plasma length $L_{p}$ and $\phi_{CEP}$ remained. This can be explained by two considerations. First, the baseline model is good enough to capture the main dependencies based on a given dataset; second, although augmentation increased dataset, it did it in a very straightforward manner without introducing new information that can be used to obtain new knowledge about data. Training on noisy data helped the model to distract from noise and focus more on the important information, and thus generalize better. Other methods of augmentation, partially sacrificing physical correctness, potentially can produce better results, which should be investigated in future works. As an example, averaging spectra for neighboring points in a parameters space to produce new points in this space can be used, but it requires reasonably good resolution in the parameter space to produce nearly correct results.

#### 4.3.4. Comparison of the Results

The results of the accuracy of the considered approaches are compared in Table 1. For each of the parameters, the use of PCA increased the accuracy in comparison with the baseline model, when training was performed for all features. The data augmentation outperforms the other two approaches in accuracy, so the relative error reduced by 15–20% for each of the parameters in comparison with the baseline model.

**Figure 5:**
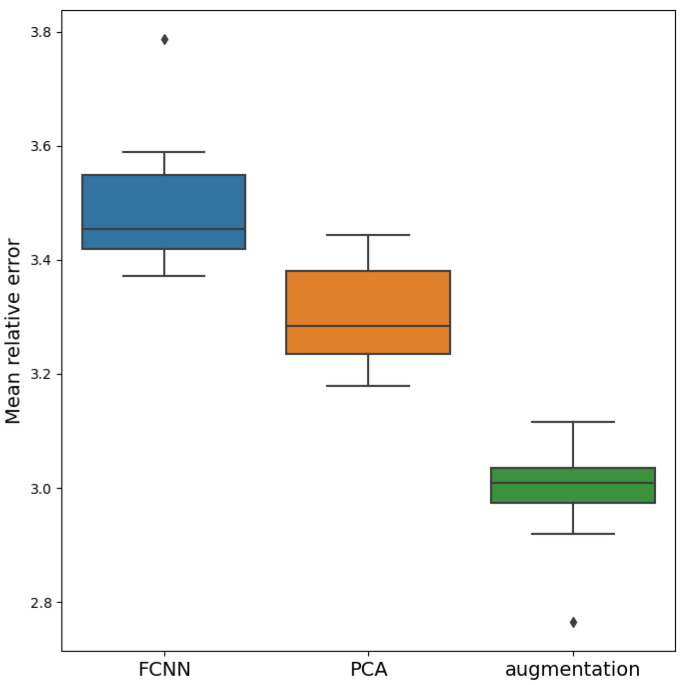
Distribution of mean relative percentage errors for 3 models: baseline model, model trained on PCA features and model trained on augmented data.

The distribution of the accuracy of the models for ten runs is shown in Figure 5. Using the two-sample Student’s *t*-test, we checked if the mean values of the obtained samples for the three considered models pairwise differ (baseline vs. PCA preprocessing vs. data augmentation). At the significance level of p=0.05, the results showed that the mean values of all three samples differ. Thus, we conclude that the use of data augmentation provides the best accuracy.

### 4.4. On the Way to Experimental Data Processing

#### 4.4.1. Basic Idea

In previous sections, all the described methods were applied to clean data, obtained directly from the numerical simulations. Although this problem itself can be of interest, for example, one can imagine tailoring numerical simulation parameters based on desired spectra, we aim at applying the proposed approach to extract relevant information from real experimental data. There are several problems that can arise during such analysis: experimental conditions may differ from that used in simulations, experimental spectra can be measured imprecisely and/or affected by additional factors or, effectively, the spectra may be overly noisy. In this paper, we want to address the latter case, namely, working with noisy data.

A priori, there is no certainty that the approaches considered earlier in relation to data obtained by numerical simulation methods will work with reasonable or even acceptable accuracy on noisy data of a physical experiment. First, we would like to see to which extent the accuracy of the parameter reconstruction deteriorates if the spectrum contains noise. Second, we want to investigate methods that can help to keep the accuracy at a reasonable level even in the presence of noise. To address this problem, we added Gaussian noise with zero mean over the entire original dataset, computed by numerical simulation. One can argue that we have already performed the same procedure while performing data augmentation. There are two main differences between working with noisy data and the data augmentation presented in the previous section. First, in the case of augmentation, we added noise only during training to improve the generalization abilities of the model, while inference was performed on clean data. In this section, noise is superimposed on both training and test samples to simulate noisy data in a real experiment. Second, in data augmentation, experiments variance of additional noise was chosen proportional to the spectral density of each individual sample. In this section, variance of the noise is chosen according to a more advanced model.

**Figure 6:**
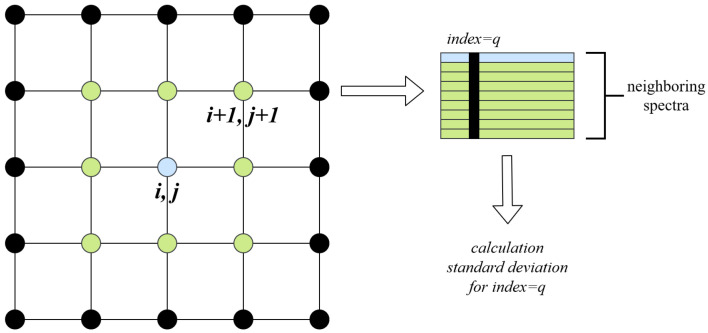
The scheme for computing the standard deviation estimate for the noise generation (the case of 2D parameter space is considered for lucidity). On the left, the blue dot represents the spectrum $S_{i,j}$ in 2D parameter space, green dots show spectra in neighbor points in parameters space $S_{i+k,j+w}$, where k,w∈[−1,0,1]. On the right, the blue line corresponds to the $S_{i,j}$ spectrum, whereas green lines depict the $S_{i+k,j+w}$ spectra. The black vertical line shows spectral density values for index *q* (indicating the frequency) that are used for standard deviation estimation.

We estimate the empirical variance of spectral intensity for each frequency separately, using the values of 26 neighbors in the 3D parameter space (and a smaller number for the points at the boundaries). The estimated variance is then used to add random distortions that are normally distributed with the estimated variance, which depends on both frequency and location in the parameter space (see Figure 6).

**Figure 7:**
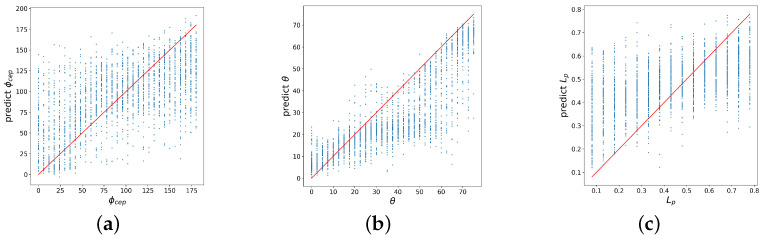
Correlation of the exact and predicted values of laser–plasma interaction parameters, reconstructed by a baseline fully connected NN trained on clean data and tested on noisy data: (**a**) carrier envelope phase, (**b**) incidence angle, (**c**) pre-plasma length.

To begin with, we evaluated the results of a model trained on clean data. We took a model trained on clean data and tested it on noisy data. We observed that the accuracy has dropped by a factor of about 4–5 (see Table 2). Figure 7 shows that the prediction accuracy has decreased significantly.

#### 4.4.2. Baseline Model

As a first step, we trained the same baseline model from Section 4.3.1 on noisy data, taking the same network architecture and parameters for training. We also tried other architectures and parameters as well, but they have not shown any significant improvement over the baseline model. The average accuracy over 10 runs is shown in Table 2. It can be seen that the relative error of reconstruction of the parameters has increased by a factor of about two. The effect of reducing model accuracy arises from two problems: the presence of noise and the difficulty of training on noisy data compared to data without noise. The first problem is clear. The second one is due to the fact that the loss surface of the model becomes much more chaotic and more complex. The initial network initialization is more important. Moreover, it can be expected that when working with data from a real experiment, the results may further degrade. Therefore, it is necessary to consider approaches that can improve accuracy. Next, we will consider approaches to simplify the learning process in one way or another.

#### 4.4.3. Employing PCA to Diminish Noise

Using PCA to reduce the number of features can also help diminish the noise [51,52]. By keeping only the components that retain most of the systematic changes in the data, we may hope to discard the components that contain noise. First of all, we tried to determine the relevant number of principal components using the same approach as before. We found that as well as for data without noise, it is reasonable to use 40 principal components.

For training, we used the same network architecture and parameters as previously with the PCA. Table 2 shows the results of applying the obtained models. Accuracy has improved notably compared to the previous experiment with the noisy data. As we expected, removing components that contained little information about the data also removed some of the noise and simplified training.

#### 4.4.4. Adding Noise to Data Gradually

Next, we tried a different approach, in which the model was first trained on clean data and then was trained on data with noise. It is important to underline here that in this section all models were tested on noisy data, where the model trained on clean data shows a poor performance. The advantage of such combined approach with training on clean/noisy data is that initial training on clean data can give a good initial guess for the weights, and following training on noisy data enhance the generalizing ability of the model. This process can be improved further if we gradually increase the variance of the noise during training and use augmentation. In this respect, for data augmentation, we applied noise with a different seed five times to a sample of the training dataset.

For this experiment, we took the baseline model and trained the network on clean data using the same network settings as for the baseline model. Next, we trained the network for 200 epochs, increasing the noise in five steps evenly. That is, the first 40 epochs took 20% of the standard deviation value for noise generation, the next 40 epochs—40%, etc. Then, the network was trained for another 50 epochs, in which the learning rate was multiplied by 0.95 each epoch.

The accuracy of this approach on noisy test data is shown in Table 2. It can be seen that the accuracy is substantially improved. As was guessed earlier, during the initial learning on clean data, the network finds a good initial approximation and then it is gradually adjusted to the noise in the data and accuracy is improved. This approach is similar to the transfer learning approach [53], which is often used in many machine learning problems, for example, computer vision. This approach can be further extended based on the experimental data. The model, pre-trained on noisy data, can be further trained on such data to adjust its parameters to real experimental data. This requires enough tagged experimental data of good quality, but we expect that this can be a useful step.

#### 4.4.5. Comparison of the Results

It can be seen that simple training of the baseline model on noisy data significantly reduces the accuracy compared to the baseline model trained on clean data. The average relative error increases by about 75–100% for each of the parameters. However, at the same time, the use of PCA for the preprocessing of features makes it possible to significantly improve the accuracy in a simple way. In this case, the accuracy of the model increases but is still 30–40% worse than the baseline without noise. Using a gradual addition of noise to the training data, as well as augmentation, allows us to approach the accuracy of models trained on data without noise. The average relative error of parameter recovery is only 5–10% worse than that in the baseline case without noise.

**Figure 8:**
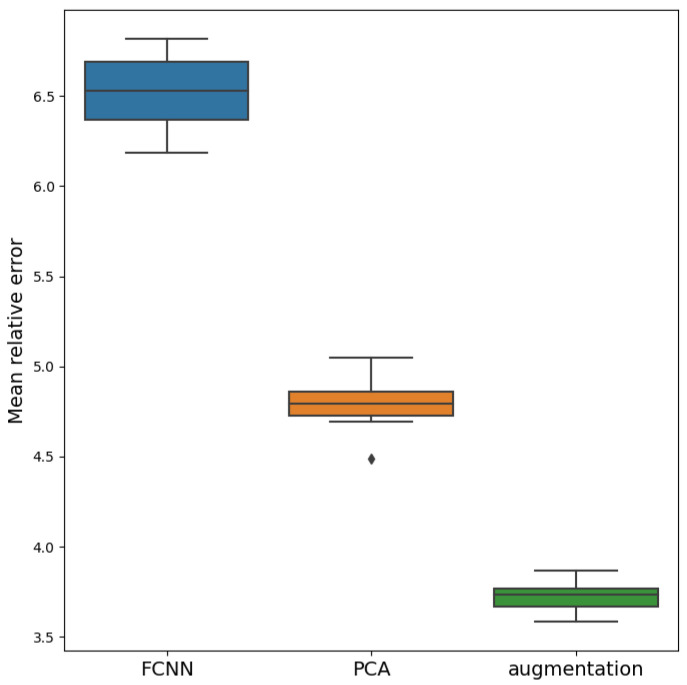
Distribution of mean relative percentage errors for 3 models trained on noisy data.

We also compared the results obtained using the paired Student’s *t*-test for the collected samples. The test at p=0.05 significance level showed that the mean values of all three samples differ. The distribution of the accuracy of the models is presented in Figure 8.

## 5. Conclusions

In this paper, we report on the development of ML-based diagnostics of laser–plasma interactions in experiments. We assess the use of NNs, trained on simulated XUV radiation spectra, to determine interaction parameters that are difficult or impossible to measure in experiments. We focus on the problem of the sensitivity of NNs to the noise in the input data or to other differences between simulated and measured spectra. First, we analyzed the possibility of solving the problem on clean data obtained from PIC simulations. Even at the baseline, the fully connected NN showed acceptable accuracy; applying additional data processing improved its quality. Principal component analysis enhanced the generalization ability of the trained model, leading to reduced mean prediction error.

We explored several approaches to the more realistic case of noisy data, which is expected to be observed in an experiment. These conditions were modeled by adding Gaussian noise with zero mean and variance characterized based on the neighboring points in parameter space. We showed that simplistic training of the baseline model on such data leads to an accuracy drop of about 75–100%, for each of the parameters, compared to the baseline model. Applying PCA preprocessing improves accuracy, but the accuracy is still 30–40% worse than that of the baseline model trained on clean data. The best result is achieved when we use superimposed noise, with an amplitude that gradually increases over the training process [17]. We observed that this approach makes it possible to reach almost the same accuracy, even if the variance of the superimposed noise is equal to the characteristic variance of the data values measured over the results for neighboring points in the parameter space. We see this as a decisive element for our further developments of ML-based diagnostics in the experiment on high-intensity laser–matter interactions. Given the generic and sophisticated nature of the considered problem, we conjecture that the considered approach, of gradually increasing noise, can be useful in the developments of ML-based diagnostics in other areas of physics.

Although we expect that the considered method of raising noise can be a good heuristic choice in similar conditions, more systematic analysis is needed, and this leads to a number of open questions. First, it is not clear how to define a universal criterion that can quantify the ability of an approach to make an ML model tolerant to noisy input data. Second, it is interesting to understand what kind of characteristics can be used to predict which approach is the most efficient in a particular project. Finding efficient ways to make ML models tolerant of systematic differences between simulation and experimental results will thus continue to excite interest.

## Figures and Tables

**Table 1:** Accuracy and coefficient of determination for the reconstruction of parameters of the laser–plasma interaction for the models trained on clean data.

Model	Metrics	$\Phi_{CEP}$	θ	$L_{p}$
Baseline	MAPE	3.823	1.781	4.890
	$R^{2}$	0.952	0.991	0.904
PCA preprocessing	MAPE	3.674	1.684	4.537
	$R^{2}$	0.956	0.992	0.928
Data augmentation	MAPE	3.233	1.493	4.236
	$R^{2}$	0.961	0.993	0.920

**Table 2:** Accuracy and coefficient of determination for reconstruction of parameters of laser–plasma interaction for the models trained on clean data.

Model	Metrics	$\Phi_{CEP}$	θ	$L_{p}$
Baseline model trained on clean data, tested on clean data	MAPE	3.823	1.781	4.890
	$R^{2}$	0.952	0.991	0.904
Baseline model trained on clean data, tested on noisy data	MAPE	18.675	13.297	18.601
	$R^{2}$	0.327	0.638	0.304
Baseline model trained on noisy data, tested on noisy data	MAPE	7.638	3.179	8.743
	$R^{2}$	0.841	0.968	0.756
PCA preprocessing	MAPE	5.385	2.434	6.537
	$R^{2}$	0.944	0.988	0.893
Adding noise to data gradually	MAPE	4.170	1.911	5.095
	$R^{2}$	0.946	0.988	0.897

## Data Availability

The data and scripts required to reproduce the numerical results may be downloaded from https://github.com/hi-chi/Machine-Learning (the relevant examples are located in the “Tight focusing” folder).
